# Clinical analysis and literature review of a complicated superior mesenteric artery stenosis with intestinal necrosis: A case report

**DOI:** 10.1097/MD.0000000000033586

**Published:** 2023-04-28

**Authors:** Binbin Zhou, Junjie Huang, Xiaobin Chen, Chen Lin, Yin Xia, Rong Jiang

**Affiliations:** a Fuzong Clinical Medical College, Fujian Medical University, Fuzhou, China; b Fujian University of Traditional Chinese Medicine, Fuzhou, China; c Department of General Surgery, 900th Hospital of Joint Logistics Support Force, Fuzhou, China; d Dongfang Hospital, Xiamen University, Fuzhou, China; e Fujian Provincial People’s Hospital, Fuzhou, China; f Department of Vascular Surgery, the Affiliated People’s Hospital of Fujian University of Traditional Chinese Medicine, Fuzhou, China.

**Keywords:** case report, intestinal adhesions, intestinal fistula, intestinal necrosis, superior mesenteric artery stenosis

## Abstract

**Rationale:**

Superior mesenteric artery (SMA) stenosis, as a common arterial disease, if coexists with other possible causes of abdominal pain, is complicated, which may require not only conservative treatment but also surgical intervention.

**Patient concerns:**

A 64-year-old male patient who was admitted to our hospital with pain located around the umbilicus and right lower quadrant for 12 hours.

**Diagnosis:**

SMA stenosis was initially diagnosed. After balloon dilatation of SMA and stent implantation, computed tomography angiography reexamination showed that the stent was migrated and the stenosis reoccurred. During the ileocecal resection and enterolysis, the necrotic bowel was found and cut open, and the intestinal fistula was found. Combined with his abdominal surgery history, the patient was diagnosed with complicated SMA stenosis with intestinal necrosis.

**Interventions:**

The balloon dilatation of SMA and stent implantation was performed. Because the stent was migrated and the stenosis reoccurred, so a balloon stent was implanted in the proximal stenosis of SMA again. The patient’s symptoms were relieved and reoccurred again. The ileocecal resection and enterolysis were performed.

**Outcomes:**

The computed tomography angiography showed that the stents were well deployed and unobstructed after 9 months follow-up.

**Lessons:**

When dealing with undetermined abdominal pain that especially has something to do with mesenteric artery ischemia, if there coexists with other possible causes of abdominal pain, we cannot only focus on vascular diseases. We should be vigilant, integrate multiple factors and their interactions to guarantee the accuracy and timeliness of diagnosis and therapy.

## 1. Introduction

The prevalence of stenosis of the superior mesenteric artery (SMA) which is common showed a gradual increase with age. The etiology mainly includes atherosclerosis or thrombosis.^[1]^ Although patients are generally asymptomatic, a developing SMA stenosis may represent an uncommon cause of chronic or acute mesenteric ischemia that results in a series of symptoms including the classical triad that only 60% of patients have at the same time, usually characteristic of post-prandial epigastric pain or abdominal distension, weight loss, and abdominal vascular murmur,^[2]^ and unexplained chronic gastroduodenal ulcer or right colitis where SMA provides blood supply.^[3]^ This literature reported an unusual case of a patient with complicated SMA stenosis with intestinal necrosis.

## 2. Case report

**Figure 1.**
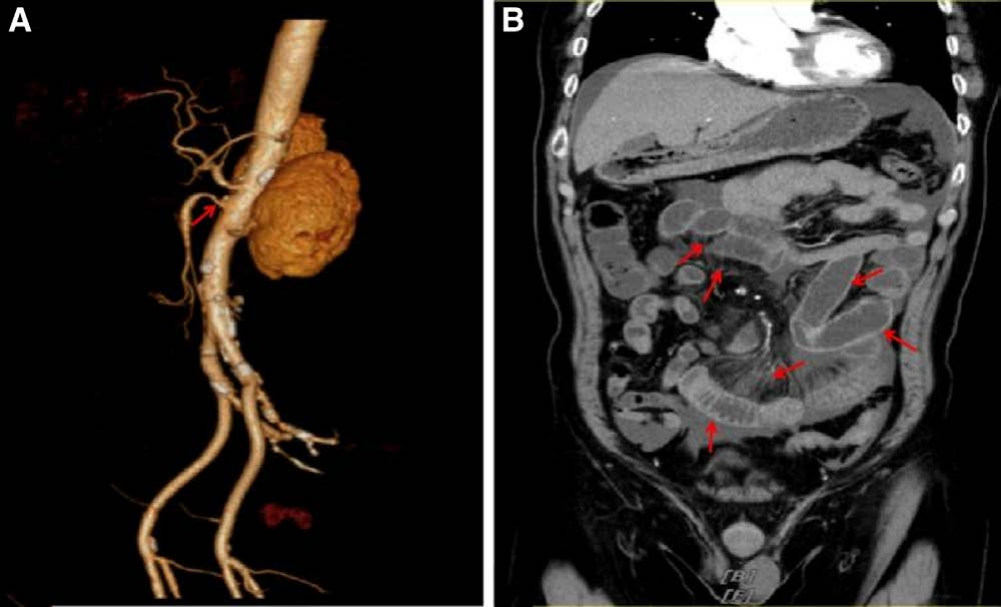
Abdominal CTA examination showing (A) stenosis at the opening of superior mesenteric artery and (B) ischemic changes in partial bowel and mesentery. CTA = computed tomography angiography.

A 64-year-old male was admitted to our hospital with pain located around the umbilicus and right lower quadrant for 12 hours. The patient suffered from pain around the umbilicus and right lower abdomen with no inducing factors 12 hours before admission, persistent and paroxysmally increase in severity, accompanied by nausea and undigested food vomiting. So he was present at our outpatient clinic, and admitted with “abdominal pain of undetermined reason.” The patient had hypertension for 2 years, and the highest blood pressure was 160/110 mm Hg. He irregularly took antihypertensive drugs and did not regularly monitor his blood pressure; Abdominal surgery was done many years ago (details unknown). Physical examination: Periumbilical and right lower abdominal tenderness was the main positive symptom, and other parts were negative. No obvious abnormality was found in the blood routine test, complete set of biochemistry, blood coagulation function, myocardial zymogram, electrocardiogram, color Doppler ultrasound of the heart, and peripheral blood vessels. The results of his abdominal computed tomography angiography (CTA) examination showed that: the soft and hard plaques at the beginning of the SMA led to secondary lumen stenosis; the partial wall of the small intestine was edematous, and the CT value of the mesentery was increased, which were considered to be the ischemic changes with pelvic and peritoneal effusion that significantly increased (Fig. 1). Admission diagnosis: SMA stenosis, ischemic enteropathy, and hypertension.

**Figure 2.**
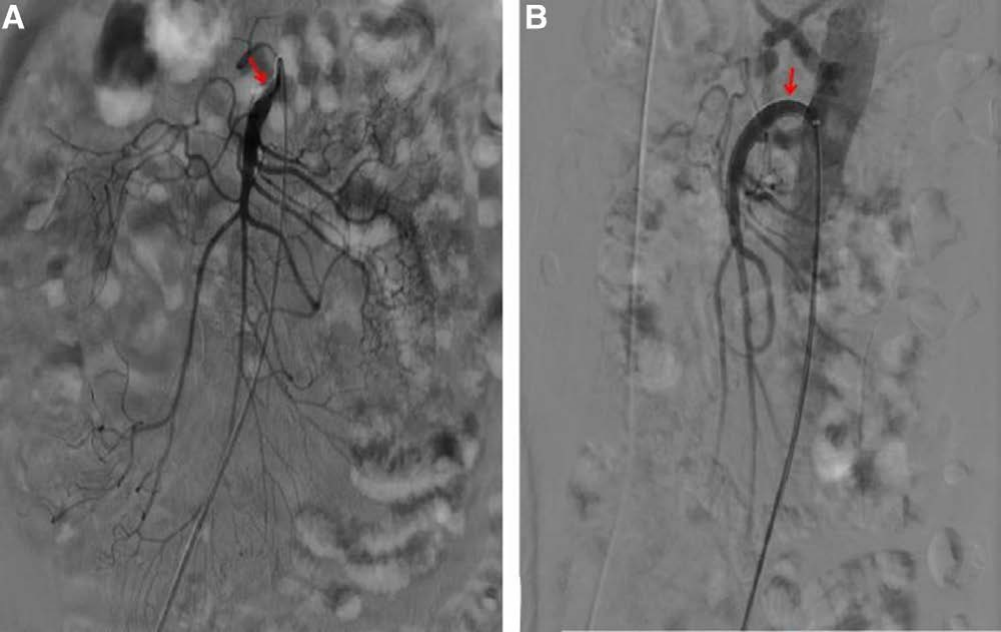
(A) Digital subtraction angiography (DSA) confirming the stenosis of superior mesenteric artery during operation. (B) As soon as 1 EV3 bare stent was implanted, the blood flow was significantly improved.

**Figure 3.**
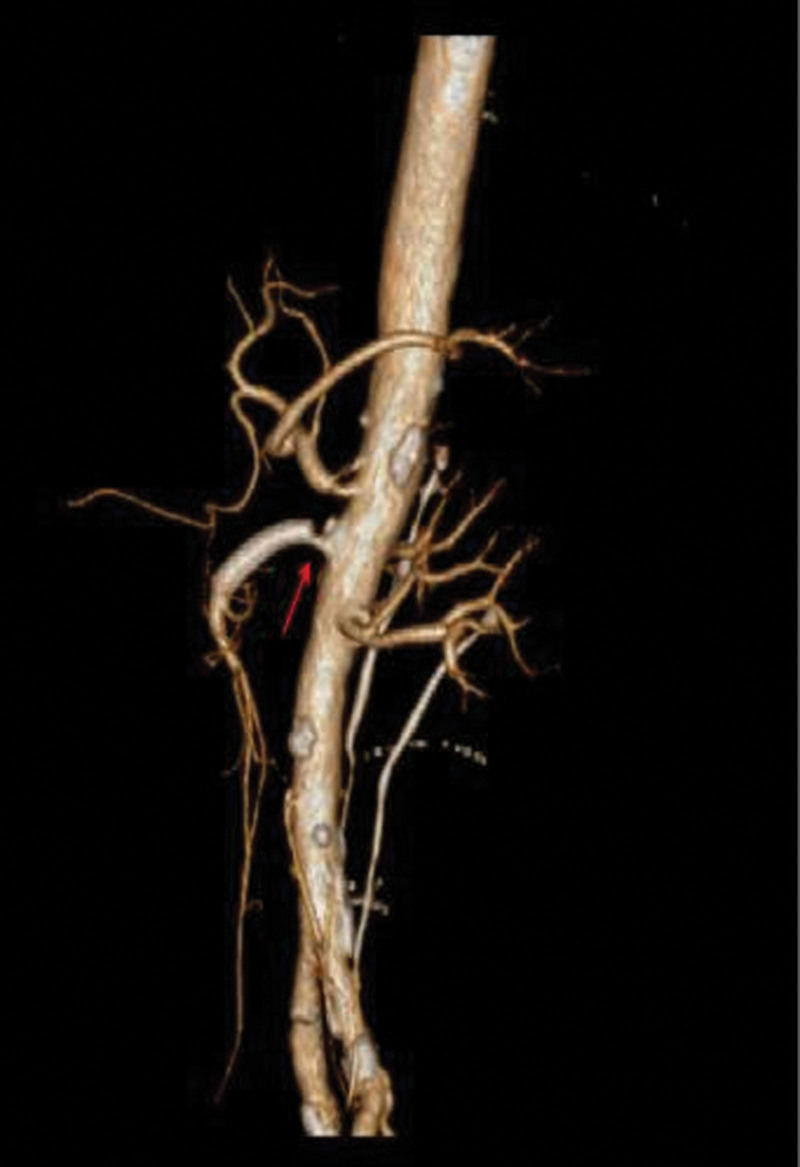
The opening of superior mesenteric artery was obviously narrowed and the stent moved to the distal end.

**Figure 4.**
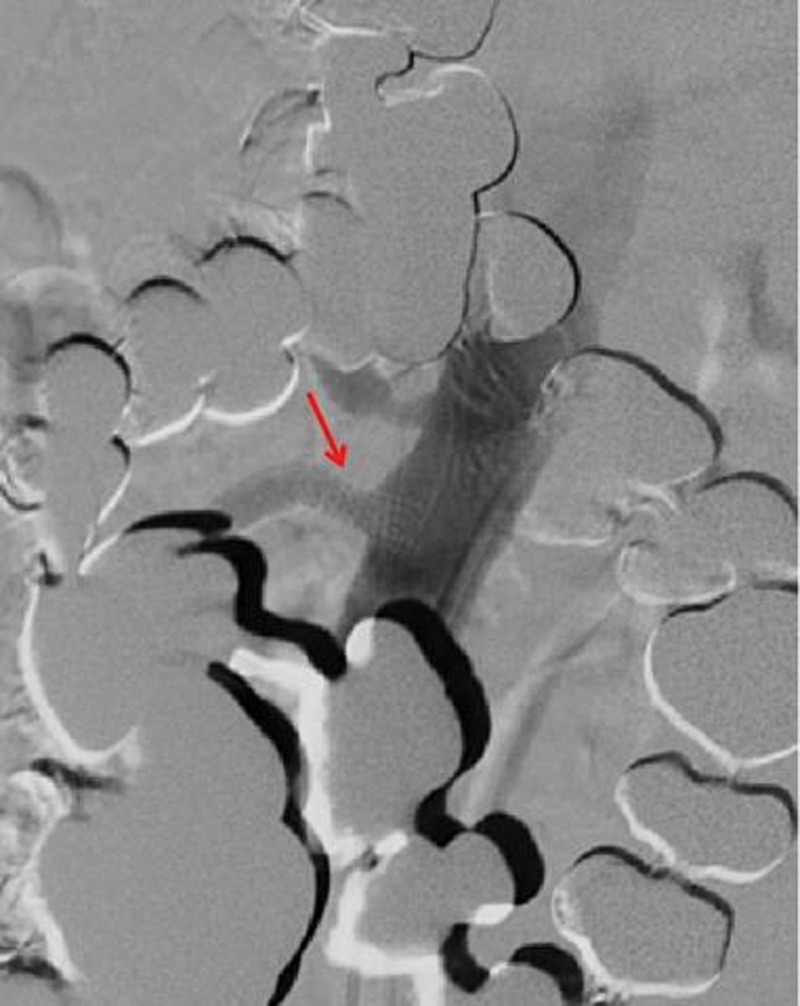
One balloon-expandable stent implanted during operation.

**Figure 5.**
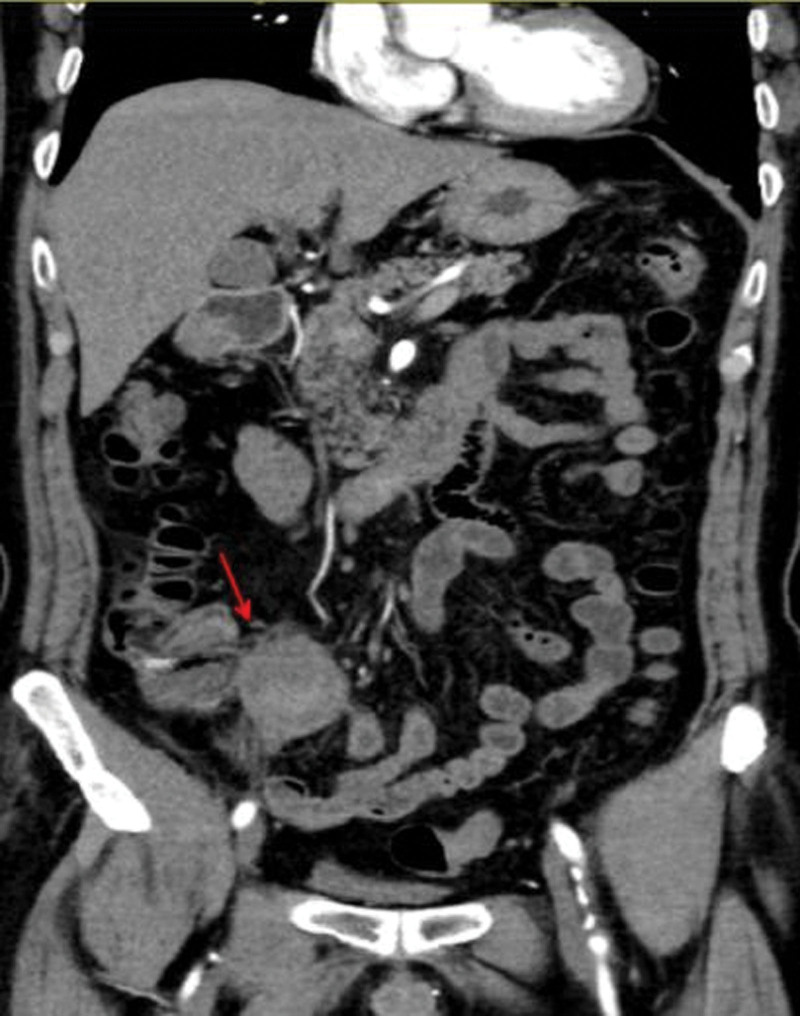
Abdominal CTA examination showing obvious intestinal edema with intestinal adhesion in the ileocecal region. CTA = computed tomography angiography.

**Figure 6.**
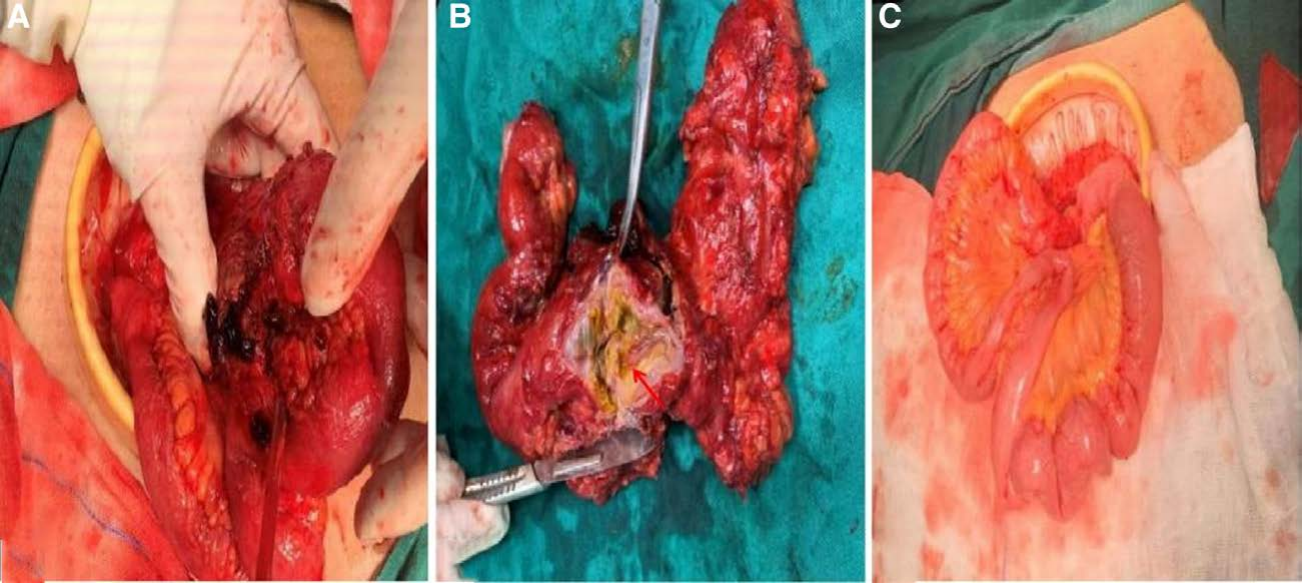
(A) Ileocecal intestinal adhesions accompanied with partial intestinal necrosis were resected, in which (B) the intestinal fistula was found. (C) The residual intestine remained normal after resection.

**Figure 7.**
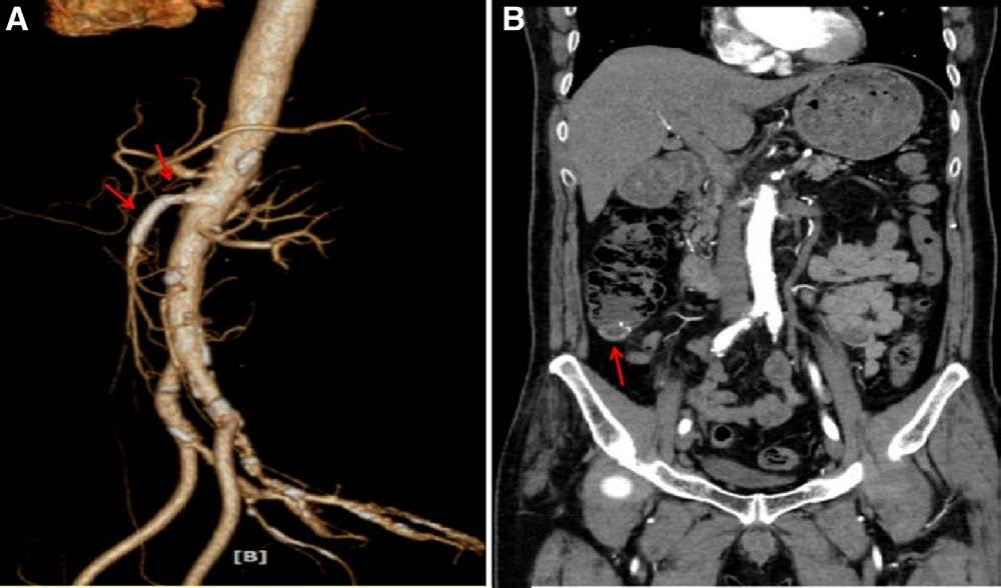
(A) Abdominal CTA reexamination showing that 9 months after operation, the stents at the opening of superior mesenteric artery were in place and the blood flow was smooth. (B) No intestinal abnormality. CTA = computed tomography angiography.

In terms of treatment, after 5 days of fasting and parenteral nutrition treatment, the patient’s symptoms of abdominal pain and abdominal distension were slightly relieved but still waxed and waned. After eliminating the relevant surgical contraindications, balloon dilatation of SMA + stent implantation was performed under local anesthesia. During the operations, it was observed that the opening of SMA was narrowed, the blood flow was slowed, and the distal artery was smooth, so an EV3 bare stent (6–30 mm, Cordis Corporation, https://cordis.com/apac/about) was implanted (Fig. 2). After operation, the abdominal symptom diminished immediately, and the patient did not complain of any other discomfort. On the 8th day after the operation, the patient began to accept a liquid diet. On the 18th day after the operation, the patient had post-prandial abdominal pain and distension again with hiccups. The only symptom was still slight tenderness in the right lower quadrant of the abdomen, and the bowel sounds were normal. The results of postoperative CTA showed that the stent was unobstructed but displaced, and the proximal opening of the SMA was narrow again (Fig. 3). Therefore, stenting of SMA was performed under local anesthesia. During the operation, a balloon-expandable stent (6–40 mm, ASAHI INTECC CO., LTD, http://www.asahi-intecc.co.jp/) was implanted at the proximal stenosis of SMA (Fig. 4). After the operation, the abdominal symptom diminished again. On the 16th day after the second operation, the patient suffered from abdominal pain in the right low quadrant and abdominal distension again after meal. After 1-week conservative treatment, such as fasting, acid inhibition, vasodilation, fluid infusion, and nutritional support, the symptom continued without change. As the abdominal CTA results implied the possibility of the ischemic necrosis of the end ileum (Fig. 5), ileocecal resection and enterolysis were performed under general anesthesia. During the operations, ileocecal intestinal adhesions and local ischemic necrosis were seen, necrotic bowel tubes were cut, and an intestinal fistula was disclosed inside the necrosis, however, no obvious abnormality was found in any other part of the small intestine in vivo (Fig. 6). With the proper perioperative support including anti-infection, fluid infusion and nutritional support for 14 days after the third operation, the patient recovered well with the well-healed incision, the good general condition, no abdominal discomfort after eating and the normal defecation, and was discharged from hospital. The results of reexamination of abdominal CTA in our hospital 9 months after the operation showed that the stents in the SMA were well deployed and unobstructed, and the ileocecal region showed postoperative changes (Fig. 7), indicating that the patient had a good prognosis.

## 3. Discussion

This literature reported that the complicated SMA stenosis with intestinal necrosis in this case was successfully treated by endovascular and surgical treatment. The features of this rare case may offer clinicians some experience in diagnosis and treatment.

The focus can be easily recognized by advanced imaging techniques such as Doppler ultrasound, CTA, magnetic resonance angiography, and digital subtraction angiography which is the golden criterion of diagnosis. The patient who was admitted with the main complaint of epigastric pain and CTA results showing a proximal stenosis of SMA with atherosclerosis was diagnosed. At the same time, CTA suggested the typical imaging manifestations of intestinal ischemia, such as the extensive expansion of the partial small intestine, enteral effusions, etc., so we considered that the patient’s abdominal pain might be caused by intestinal ischemia induced by the proximal lesion of SMA.

When it comes to treatment, generally speaking, asymptomatic patients are always under conservative therapy. For symptomatic patients, surgery is mainly considered, including open operation, and endovascular treatment which is usually the prime choice because of its high clinical effectiveness and few complications. However, because of the difficulty of exact assessment of the progress of intestinal necrosis, no matter which treatment is taken, there still exists a probability of reoperation.^[4]^ The patient’s CTA showed that the stenosis at the opening of the SMA was about 2.51 mm in diameter, 14.3 mm in length, and 64.14% in degree, and an atherosclerotic plaque was formed at the opening, which implied a high risk of arterial embolism. The above imaging features and ineffective conservative treatment indicated surgery. After our communication with the patient and his family, an agreement on endovascular treatment (including balloon expansion, coil embolization, stent implantation, etc) was achieved. Among the above methods of endovascular intervention, simple balloon dilation has a high rate of recurrence and limited effectiveness. The increasing experience in the clinical application of intravascular stent, the improvement of stent configuration, and the refinement of the delivery catheter have improved the safety and efficacy of stent implantation in treating visceral vascular stenosis.^[5]^ However, there is still no consensus on the selection of stents. Recently, self-expanding bare stents that have better compliance and more smooth deliver system, with the advantage of tolerating the curvature that is usually located in the proximal opening of SMA are the most widely used in mesenteric artery diseases^[6]^ to maintain the original arterial radian, prevent new damage on intima, and also ensure the patency of branches. Despite the relatively poor radical strength, it is considered to be enough to render support. Therefore, a self-expanding bare stent was chosen, and digital subtraction angiography during the operation indicated that the stenosis disappeared, the proximal vascular diameter was enlarged to approximately 7.06 mm and the blood flow was significantly improved as soon as the stent was implanted.

Unfortunately, not so long after the operation, the patient had a sensation of abdominal discomfort again. The abdominal CTA showed proximal stenosis of SMA reoccurred, and the stent moved to the distal end as a whole. After comprehensive consideration, it was convinced that the restenosis due to the stent displacement should induce intestinal ischemia and abdominal discomfort. It is generally believed that stent displacement which means that the stent moves more than 10 mm or clinical symptoms appear that need treatment, has an incidence of about 2 to 3%,^[7]^ probably higher. The causes of stent displacement mainly include vascular factors (lesion, blood flow, etc) and stent factors (stent materials, stent deployment operations, etc).^[8]^ Since the patient’s lesion was located at the opening, the center of the stent was positioned a little toward the distal end to prevent the proximal end of the stent from entering the abdominal aorta. What is more, the atherosclerotic plaque at the opening may affect the full deployment and adherence of the stent, which may be another reason for stent displacement. Optional management for stent displacement includes retrieval, re-position, conservative treatment, and surgical removal. Due to the ineffective conservative treatment and the irretrievability of the stent, a second stent implantation was performed and a balloon-expandable stent that was of better accuracy of positioning was implanted at the restenosis of SMA.

Despite the continually decreasing inflammatory indicators of the patient, the steady D-dimer, and the reexamination of CTA indicating that the intestinal ischemia and edema were relieved and the inflammation was more absorbed than before, which may owe to the intervention treatments, the patient still had post-prandial abdominal symptoms, which were limited to the right lower abdomen. Combined with the patient’s previous surgery in the right lower abdomen, it was speculated that the intestinal adhesion in this area due to the previous surgery might have caused localized ischemia or even necrosis. It was reported that the incidence of intestinal adhesion after abdominal surgery was about 63 to 97%,^[9]^ however, the mechanism of intestinal adhesion is still unclear. A large amount of fibrin and plasminogen activator inhibitors are considered to be secreted in the operation zone by the intestinal walls that are affected by the stimuli including surgical trauma, infection during operation, resulting in the imbalance of fibrin deposition and degradation, and finally intestinal adhesion. Compared with the normal intestines, the ability of peristalsis and expansion of the adhesive intestines which are excessively distorted obviously weakens, and it is difficult for food to pass through, which may lead to incomplete intestinal obstruction. What is more, the increasing pressure of local intestinal tubes may occlude intestinal vein and lymphatic circulation, swell the intestinal wall, and then compress arterioles and capillaries, leading to insufficient perfusion, and then intestinal ischemia that is usually combined with continuous inflammatory stimulation, eventually intestinal necrosis or intestinal fistula. So far, there is no effective treatment for intestinal adhesion. The patient’s intestinal adhesions secondary to the operation he underwent for ileocecal lesions many years ago, accompanied by SMA stenosis, may aggravate ileocecal ischemia. As conservative treatment did not work well, the patient underwent laparotomy. During the operation, the necrosis of the adhesive intestine and the formation of an intestinal fistula at the adhesive site were confirmed, so ileocecal resection and enterolysis were done. After the operation, the patient’s abdominal symptoms dissolved, and a 9-month follow-up indicated a favorable prognosis.

## 4. Conclusion

In conclusion, when dealing with undetermined abdominal pain that especially has something to do with mesenteric artery ischemia, if there coexists with other possible causes of abdominal pain (such as history of abdominal surgery, early pancreatic cancer, lymph node metastasis at the root of the mesentery, etc), we cannot only focus on vascular diseases. The abdominal symptoms of patients may not only be related to local lesions, but also induced by mesenteric artery ischemia, and can remain the same or even worsen after the mesenteric artery problem is solved,^[5]^ which possibly explains the change in our patient after the endovascular intervention. Therefore, for the sake of preventing more serious complications such as acute intestinal necrosis, and ischemia-reperfusion injury,^[10]^ the treatment algorithm, conservative or surgical treatment, simultaneous or elective surgery, should be timely and accurately determined, according to the continual and comprehensive preoperative assessment of the degree of intestinal ischemia, especially the areas which are combined with other lesions, which consists of the imaging and laboratory examinations (CTA, D-dimer, serum lactic acid, peripheral blood leukocytes, etc)^[11]^ and more importantly, the changes of abdominal symptoms. We should be vigilant and integrate multiple factors and their interactions to guarantee the accuracy and timeliness of diagnosis and therapy.

## Acknowledgments

The authors would like to thank all colleagues for data collection from the Department of General Surgery, 900th Hospital of Joint Logistics Support Force.

## Author contributions

**Conceptualization:** Binbin Zhou, Xiaobin Chen, Chen Lin, Rong Jiang, Yin Xia.

**Data curation:** Junjie Huang, Xiaobin Chen.

**Formal analysis:** Junjie Huang, Yin Xia.

**Resources:** Junjie Huang, Xiaobin Chen.

**Writing – original draft:** Binbin Zhou.

**Writing – review & editing:** Chen Lin, Rong Jiang, Yin Xia.
